# Caries prevalence of the first permanent molar and associated factors among second-grade students in Xiangyun of Yunnan, China: A cross-sectional study

**DOI:** 10.3389/fped.2022.946176

**Published:** 2022-09-29

**Authors:** Mingshan Liu, Xiaoqin Xu, Qianqian Song, Hongmei Zhang, Fang Zhang, Guangyun Lai

**Affiliations:** ^1^Department of Stomatology, People's Hospital of Xiangyun Affiliated to Dali University, Dali, China; ^2^Department of Pediatric Dentistry, Shanghai Ninth People's Hospital, Shanghai Jiao Tong University School of Medicine, College of Stomatology, Shanghai Jiao Tong University, National Center for Stomatology, National Clinical Research Center for Oral Diseases, Shanghai Key Laboratory of Stomatology, Shanghai, China

**Keywords:** first permanent molar, dental caries, prevalence, caries associated factors, epidemiological study

## Abstract

**Purpose:**

This study aimed to explore the caries prevalence of the first permanent molar (FPM) and the associated factors among second-grade students in Xiangyun of Yunnan, China.

**Materials and methods:**

A cross-sectional survey was conducted in Xiangcheng Town, Xiangyun County, China, from September to November 2020. The methodology recommended by WHO was used for the dental examination. All the parents were required to complete a questionnaire to collect information on children's family background, dietary habits, oral health behaviors and parental oral health-related knowledge. The tests of significance used were the chi-square test. The association between dental caries prevalence and its associated factors was investigated using logistic regression analysis. Statistical significance was set at *P* < 0.05.

**Results:**

Data of 1,295 second graders consisting of 665 males and 630 females were analyzed. The caries prevalence of FPM was 47.6%, and the mean DMFT was 1.11 ± 1.394 in this sample. Among all the children with caries, the filling rate is meager, 2.6%. There were statistically significant differences in the caries prevalence of FPM among age groups. No significant difference existed between the sexes. Logistic regression analysis showed that the most significantly associated factors were: consuming desserts at least once a day, no fluoride varnish application experience, worse parental perception of children's oral health status, and incorrect parental knowledge of whether decayed primary teeth need treatment.

**Conclusion:**

Caries prevalence of FPM among second-grade schoolchildren in Xiangyun was considerably higher than the average prevalence nationwide in China. As China aims to reduce dental caries to <25% of 12-year-old children in 2030, the local government of remote regions like Xiangyun needs to do much more to achieve this goal. Results of this study could provide a framework for ongoing and future public oral health programs and policy planning in Xiangyun, with particular attention to early preventive and therapeutic measures.

## Introduction

Dental caries is a biofilm-mediated, multifactorial, dynamic disease that destroys dental hard tissues (1). Sugar intake is the most important dietary factor in dental caries development (2). Dental caries and its sequelae can evoke pain and infection and affect children's physically and psychologically life quality without proper treatment (3, 4). Besides, the treatment of dental caries can last a lifetime and impose a significant socioeconomic burden on both individuals and health care systems (5, 6). Despite the significant achievement in prevention, dental caries is still one of the most common chronic diseases affecting children worldwide (1). Among American youth aged 2–19 years, the caries prevalence in primary or permanent teeth was 45.8% (7). According to the 4th Chinese National Oral Health Survey, caries prevalence has increased over the past 10 years in children aged 5 and 12 years (8).

The first permanent molar (FPM), an essential tooth in the dental arch, typically erupts at 6–7 years of age. Due to the morphological and functional characteristics combined with environmental factors, FPM is more vulnerable to caries than other teeth (9, 10). FPM is at serious risk of developing caries in the years immediately after the eruption (11, 12). Furthermore, significant associations were found between caries in the first permanent molar and caries in other permanent teeth (13). Except for chewing food, FPM is vital for maintaining the face's vertical dimension and plays a critical role in occlusion formation (14). It participates in the maxillary growth and physiology of the mandibular system (15). Hence, the early loss of FPM due to caries impacts individuals' future oral and dental health.

In order to avoid the adverse effects of caries in FPM, China launched a National Oral Health Comprehensive Intervention Program in 2008, which provides a series of measures for oral health promotion, including enhanced oral health education, and pit and fissure sealants of FPM for schoolchildren. Based on this program and the national oral health survey, the children's caries prevalence of FPM in different age groups has been investigated. For example, the average prevalence of dental caries on FPM among 6–8-year-old children in Zhejiang, a well-developed province in southeast China, increased from 20.4 to 29.0% between 2013 and 2017 (12). However, the available evidence shows caries is modulated by behavioral, social-economical, and environmental factors (1). As China is geographically vast, the economic level of each region varies greatly, and people exhibit different dietary habits; children's oral health status from different regions may vary. Until now, epidemiological data on the caries prevalence of FPM in children from remote and rural areas of China are rare. Thus, this study aimed to investigate the caries prevalence of FPM and explore associated factors among second-grade students in Xiangyun of Yunnan, China.

## Materials and methods

### Ethical considerations

This cross-sectional study was implemented in coordination with the National Oral Health Comprehensive Intervention Program for Children in China, conducted in Xiangcheng Town, Xiangyun county, from September to November 2020. The Ethics Committee of the People's Hospital of Xiangyun approved the survey protocol (No. 2020069).

### Study population

The minimum sample size was calculated using Power Analysis & Sample Size (PASS) software 16.0 with a 95% confidence level, a 0.05 margin of error, a 29% reported caries prevalence of FPM (12), and a 20% additional sample size to compensate for the possible sample loss. Finally, the minimum sample size needed was 418. According to the information provided by the Educational Organization of Xiangyun County, the number of second-grade schoolchildren from all the four primary schools in Xiangcheng Town was around 1,500 in 2020, which is much larger than the minimum sample size needed.

### Inclusion and exclusion criteria

The inclusion and exclusion criteria were applied to students participating in this study and their legal guardians/parents. We recruited all the second-grade schoolchildren. The exclusion criteria were: students unable to cooperate with the examiner, students with systemic diseases or mental disorders, the legal guardian unwilling to sign the informed consent, and incomplete information in the questionnaire filled by the guardians.

### Date collection

#### Clinical examination

The dental examination was conducted using the methodology proposed by WHO, 2013 (16). All the examiners with working experience for at least 3 years were from the Department of Stomatology of the People's Hospital of Xiangyun. They received theoretical and clinical operation training before the survey. The inter-examiner Kappa values were over 0.85, indicating high levels of inter-examiner consistency. The trained dentists examined children with a plane mouth mirror and a probe under artificial light in the classroom.

#### Questionnaire survey

Questionnaires modified based on questions used in the 4th Chinese National Oral Health Survey (8) were distributed and collected by teachers in each school who received unified training before the initiation of the field investigation. With the consent form, the parents or guardians were asked to complete the questionnaire the day before the clinical examination of their children. The questionnaire was composed of four parts:

- Family background (single child or not, the primary caregiver for children; the parental education level, etc.)
- Oral health behaviors (tooth brushing frequency, dental floss usage, etc.)
- Dietary habits (consumption of sugar, drinks, candy/chocolate, etc.)
- Oral health assessment, dental knowledge, experience, and attitude (the importance of oral health in the quality of life, the treatment necessity for decayed primary teeth, the fluoride application, etc.)

### Data analysis

Categorical variables were expressed as numbers and percentages (%). The DMFT scores were exhibited in mean ± SD. The tests of significance used were the chi-square test. The association between dental caries prevalence and variables with statistical significance was determined using logistic regression analysis. All data were analyzed using SPSS Statistics software Version 25.0 (IBM, Chicago, IL, USA). Statistical significance was set at *P* < 0.05.

## Results

In general, 1,587 children aged 7–9 years, consisting of 837 males and 750 females, received the dental examination. However, 292 children were excluded due to the lack of consent from guardians (*n* = 287) and insufficient data (*n* = 5), such as the absence of family income information in the questionnaire. Finally, data from 665 males and 630 females were included for statistical analysis in this study (Table 1).

**Table 1:** Prevalence of caries, DMFT, filling rate and pit and fissure sealant rate of first permanent molar according to different children's characteristics (*N* = 1,295).

**Variables**	**Subjects**	***N***	**Prevalence**	**DMFT (mean ±SD)**	**Filling (%)**	**PFS (%)**
**Sex**						
Male	665	301	45.3%	1.05 + 1.374	8 (2.7%)	2 (0.3%)
Female	630	316	50.2%	1.18 + 1.413	8 (2.5%)	2 (0.3%)
**Age**						
7	388	164	42.3%^*^	0.94 + 1.311^*^	3 (1.8%)	0 (0%)
8	661	325	49.2%	1.15 + 1.405	9 (2.7%)	3 (0.5%)
9	246	128	52.0%	1.28 + 1.470	4 (3.1%)	1 (0.4%)
**Single child**						
Yes	327	156	47.7%	1.09 + 1.379	3 (1.9%)	3 (0.9%)^*^
No	968	461	47.6%	1.12 + 1.400	13 (2.8%)	1 (0.1%)
**Ethnicity**						
Han	1,023	489	47.8%	1.09 + 1.373	12 (2.5%)	2 (0.2%)
Others	272	128	47.1%	1.18 + 1.472	4 (3.1%)	2 (0.7%)

* Statistically significant at P < 0.05.

### Caries prevalence of FPM

The caries prevalence of FPM was 47.6%, and the mean DMFT was 1.11 ± 1.394 in this sample. There were significant differences in caries prevalence and DMFT between age groups (*P* = 0.03 and 0.011, respectively). Additionally, the caries prevalence and DMFT of the mandibular FPM were significantly higher than the maxillary FPM (Table 2, *P* < 0.0001). The prevalence was slightly higher in females (50.2%) than in males (45.3%), but the difference was statistically insignificant. The caries prevalence of FPM and DMFT were similar between non-single children (47.6%) and single children (47.7%). Han ethnic children showed a comparable prevalence to other ethnic children (47.8 vs 47.1%).

**Table 2:** Prevalence of caries, DMFT of first permanent molar in different locations (*N* = 1,295).

**Variables**	**Subjects**	***N***	**Prevalence**	***P*-value**	**DMFT (mean ±SD)**	***P*-value**
Maxillary	1,295	288	22.2%	<0.0001^*^	0.35 ± 0.699	<0.0001^*^
Mandibular	1,295	584	45.1%		0.76 ± 0.895	

* Statistically significant at P < 0.05.

### Filling and pit and fissure rate of FPM

Among all the children with caries, the filling rate is meager, 2.6%. The differences between sex groups, ethnic groups and single child or not groups were statistically insignificant. The pit and fissure sealant rate of FPM was 0.3%, equal between males and females. No child received pit and fissure sealant in the 7-year-old age group.

### Factors associated with caries of FPM

According to the questionnaire survey, most of the children (90.9%) were taken care of by their parents (Table 3). Both the parental education and family income level had no impact on the caries prevalence of FPM (*P* > 0.05).

**Table 3:** Prevalence of dental caries of first permanent molar and associated factors according to the questionnaire (*N* = 1,295).

**Variables**	**Subjects**	***N***	**Prevalence (%)**	**Chi-square value**	***P*-value**
**Family factors**					
**Caregiver**					
Parents	1,177	558	47.4%	0.289	0.591
Others	118	59	50.0%		
**Mother**'**s education (highest level achieved)**					
Less than high school	587	283	48.2%	0.714	0.7
High school diploma	335	153	45.7%		
College and above	373	181	48.5%		
**Father**'**s education (highest level achieved)**					
Less than high school	599	284	47.4%	0.118	0.943
High school diploma	324	153	47.2%		
College and above	372	180	48.4%		
**Monthly family income (Yuan)**					
<6,000	784	376	48%	0.177	0.915
≥6,000 and <12,000	347	162	46.7%		
≥12,000	164	79	48.2%		
**Oral health behavior**					
**Frequency of tooth brushing**					
≥Twice a day	695	328	47.2%	2.754	0.252
=Once a day	534	251	47.0%		
<Once a day	66	38	57.6%		
**Parents help children brush teeth**					
Everyday	51	30	58.8%	2.888	0.236
Occasionally	850	405	47.6%		
Never	394	182	46.2%		
**Use dental floss**					
No	1,147	557	48.6%	3.381	0.066
Yes	148	60	40.5%		
**Dietary habits**					
**Frequency of desserts**					
Occasionally or never	1,021	456	44.7%	17.210	<0.0001^*^
≥Once a day	274	161	58.8%		
**Frequency of sweet drinks**					
Occasionally or never	1,212	576	47.5%	0.109	0.741
≥Once a day	83	41	49.4%		
**Frequency of candy/chocolate**					
Occasionally or never	1,104	514	46.6%	3.544	0.06
≥Once a day	191	103	53.9%		
**Snacks before bed without toothbrushing**					
Occasionally or never	1,202	563	46.8%	4.361	0.037^*^
Often	93	54	58.1%		
**Oral health assessment, dental knowledge, experience, and attitude**					
**Application of fluoride varnish**					
No	826	415	50.2%	6.168	0.013^*^
Yes	469	202	43.1%		
**History of dental visit**					
No	380	169	44.5%	2.168	0.141
Yes	915	448	49%		
**Parental perceptions of children**'**s oral health status**					
Good or fair	1,181	549	46.5%	7.221	0.007^*^
Poor or extremely poor	114	68	59.6%		
**Oral health is important to life**					
Agree	1,243	589	47.4%	0.835	0.361
Disagree/unclear	52	28	53.8%		
**Knowing the 6-year molars**					
Yes	385	177	46%	0.613	0.434
No	910	440	48.4%		
**Decayed primary teeth need no treatment**					
Disagree	1,032	471	45.6%	8.191	0.004^*^
Agree/unclear	263	146	55.5%		
**Consuming too much sugar correlates to increased caries risk**					
Agree	1,262	601	47.6%	0.010	0.922
Disagree/unclear	33	16	48.5%		

* Statistically significant at P < 0.05.

While 53.7% of the children brushed their teeth at least twice a day, 5.1% brushed their teeth less than once a day. There was no significant difference in the caries prevalence of FPM among children with different toothbrushing frequencies (*P* = 0.252). In addition, 69.6% of parents helped children brush their teeth in daily life. Only 11.4% of the children used dental floss and showed a lower caries prevalence of FPM than children who did not use it without significant differences (40.5 vs 48.6%, *P* = 0.066).

The dietary habits survey showed that 21.2% of the children (*n* = 274) ate desserts at least once a day and had a significantly higher caries prevalence of FPM (58.8%) than children who occasionally or never ate dessert (44.7%). Only 83 children consumed sweet drinks, and 191 ate candy/chocolate at least once daily. Although children with consumption of sweet drinks (caries prevalence 49.4%) and candy/chocolate at least once a day (caries prevalence 53.9%) were likely to develop caries of FPM, there were no significant differences between groups. Moreover, 93 students often ate snacks before bed without toothbrushing and showed a significantly higher prevalence than children who occasionally or never did (58.1 vs 46.8%, *P* = 0.037).

The caries prevalence of PFM in children with fluoride varnish application experience (43.1%) was much lower than that in children without fluoride varnish application (50.2%). Among all the children, 29.3% of children who had never seen a dentist had a caries prevalence of 44.5%, while the children with dental visit history had a prevalence of 49%. Most children's (91.2%) parents believed that their children's oral health was very good or fair, and the caries rate of these children was significantly lower (46.5%, *P* = 0.007). There were 263 children's parents who were unsure whether decayed primary teeth needed treatment or believed primary teeth did not need treatment. Children whose parents disagreed with primary teeth that do not need treatment had a much lower caries prevalence (45.6%, *P* = 0.004). Only 29.7% of children's parents knew the 6-year molars.

As Table 4 demonstrated, frequency of desserts, fluoride varnish application experience, parental perceptions of children's oral health status, and parental knowledge of whether decayed primary teeth need no treatment were the most critical factor for caries in FPM in this sample, with OR values of 1.838, 1.319, 1.716, and 1.379, respectively.

**Table 4:** Logistic regression analysis of factors associated with the prevalence of dental caries in first permanent molars.

**Variables**	**B**	**SE**	**Wald**	***P*-value**	**OR**
Frequency of desserts	0.609	0.14	19.01	<0.0001^*^	1.838 (1.398–2.416)
Snacks before bed without toothbrushing	0.367	0.225	2.662	0.103	1.444 (0.929–2.244)
Application experience of fluoride varnish	0.277	0.119	5.438	0.02^*^	1.319 (1.045–1.665)
Parental perceptions of children's oral health status	0.54	0.205	6.938	0.008^*^	1.716 (1.148–2.565)
Decayed primary teeth need no treatment	0.321	0.143	5.066	0.024^*^	1.379 (1.042–1.824)

B, regression co-efficient; SE, standard error; Wald, a chi-square value; P, significant level; OR, odds ratios.

* Statistically significant at P < 0.05.

### Caries risk factors associated with mothers' education level and family income

Although parental education and the family income level had no impact on the caries prevalence of FPM, statistical analysis showed that some children's oral-health behaviors and parental knowledge were influenced by mothers' education or family income level (Tables 5, 6). When mothers' education levels were higher, they would know the 6-year molars and the necessity of treating decayed primary teeth (*P* = 0.008; *P* < 0.0001); their children were more likely to brush their teeth twice a day and receive fluoride varnishes. Similarly, children from higher-income families tended to brush their teeth twice a day and receive fluoride varnishes. However, children's consumption frequency of sweet drinks and candy was higher when their mothers' education levels were higher.

**Table 5:** Parental education level and different variables (*N* = 1,295).

**Variables**	**Mothers' education level**			**Chi-square value**	***P*-value**
	**Middle school or below**	**High school**	**College and above**		
**Frequency of tooth brushing**					
≥Twice a day	271 (46.2%)	184 (54.9%)	240 (64.3%)	31.803	<0.0001^*^
=Once a day	280 (47.7%)	132 (39.4%)	122 (32.7%)		
<Once a day	36 (6.1%)	19 (5.7%)	11 (2.9%)		
**Frequency of desserts**					
Occasionally or never	475 (80.9%)	260 (77.6%)	286 (76.7%)	2.873	0.238
≥Once a day	112 (19.1%)	75 (22.4%)	87 (23.3%)		
**Frequency of sweet drinks**					
Occasionally or never	556 (94.7%)	313 (93.4%)	343 (92%)	2.919	0.232
≥Once a day	31 (5.3%)	22 (6.6%)	30 (8%)		
**Frequency of candy/chocolate**					
Occasionally or never	517 (88.1%)	288 (86%)	299 (80.2%)	11.547	0.003^*^
≥Once a day	70 (11.9%)	47 (14%)	74 (19.8%)		
**Snacks before bed without toothbrushing**					
Occasionally or never	531 (90.5%)	318 (5.1%)	353 (94.6%)	8.982	0.011^*^
Often	56 (9.5%)	17 (94.9%)	20 (5.4%)		
**Application of fluoride varnish**					
No	439 (74.8%)	214 (63.9%)	173 (46.4%)	79.672	<0.0001^*^
Yes	148 (25.2%)	121 (36.1%)	200 (53.6%)		
**Know the 6 year molars**					
Yes	154 (26.2%)	98 (29.3%)	133 (35.7%)	9.740	0.008^*^
No	433 (73.8%)	237 (70.7%)	240 (64.3%)		
**Decayed primary teeth need no treatment**					
Disagree	429 (73.1%)	266 (79.4%)	337 (90.3%)	42.030	<0.0001^*^
Agree/unclear	158 (26.9%)	69 (20.6%)	36 (9.7%)		
**Consuming too much sugar correlates to increased caries risk**					
Agree	568 (96.8%)	328 (97.9%)	366 (98.1%)	2.082	0.353
Disagree/unclear	19 (3.2%)	7 (2.1%)	7 (1.9%)		

* Statistically significant at P < 0.05.

**Table 6:** Monthly family income and different variables (*N* = 1,295).

**Variables**	**Monthly family income (Yuan)**			**Chi-square value**	***P*-value**
	**<6,000**	**≥6,000 and <12,000**	**≥12,000**		
**Frequency of tooth brushing**						
≥Twice a day	388 (49.5%)	202 (58.2%)	105 (64%)	15.499	0.004^*^
=Once a day	352 (44.9%)	129 (37.2%)	53 (32.3%)		
<Once a day	44 (5.6%)	16 (4.6%)	6 (3.7%)		
**Frequency of desserts**						
Occasionally or never	618 (78.8%)	270 (77.8%)	133 (81.1%)	0.722	0.697
≥Once a day	166 (21.2%)	77 (22.2%)	31 (18.9%)		
**Frequency of sweet drinks**						
Occasionally or never	747 (95.3%)	316 (91.1%)	149 (90.9%)	9.467	0.009^*^
≥Once a day	37 (4.7%)	31 (8.9%)	15 (9.1%)		
**Frequency of candy/chocolate**						
Occasionally or never	685 (87.4%)	279 (80.4%)	140 (85.4%)	9.293	0.01^*^
≥Once a day	99 (12.6%)	68 (19.6%)	24 (14.6%)		
**Snacks before bed without toothbrushing**						
Occasionally or never	721 (92%)	324 (93.4%)	157 (95.7%)	3.106	0.212
Often	63 (8%)	23 (6.6%)	7 (4.3%)		
**Application of fluoride varnish**						
No	535 (68.2%)	205 (59.1%)	86 (52.4%)	19.203	<0.0001^*^
Yes	249 (31.8%)	142 (40.9%)	78 (47.6%)		
**Know the 6 year molars**						
Yes	224 (28.6%)	114 (32.9%)	47 (28.7%)	2.214	0.331
No	560 (71.4%)	233 (67.1%)	117 (71.3%)		
**Decayed primary teeth need no treatment**						
Disagree	582 (74.2%)	303 (87.3%)	147 (89.6%)	36.918	<0.0001^*^
Agree/unclear	202 (25.8%)	44 (12.7%)	17 (10.4%)		
**Consuming too much sugar correlates to increased caries risk**						
Agree	760 (96.9%)	340 (98%)	162 (98.8%)	2.391	0.303
Disagree/unclear	24 (3.1%)	7 (2%)	2 (1.2%)		

* Statistically significant at P < 0.05.

## Discussion

Since the caries level of the 12-year-old age group is recommended by the WHO to assess the dental caries status of school-age children (17), many countries and regions have focused on the caries level of children aged 12 (18–21). However, the FPM, which many parents often ignore, can decay in the first 2–3 years after the eruption. Thus, paying attention to the caries status of FPM in children aged 7–9 is necessary. This cross-sectional study assessed the caries prevalence and associated factors of FPM among second-grade schoolchildren in Xiangyun County of Yunnan Province, China. The findings in this study are of great significance for caries prevention and treatment of FPM and for promoting overall oral health for local children.

Generally, this study showed a significant association between the caries prevalence of FPM and age. Nearly half of the second-grade schoolchildren (47.6%) in Xiangyun County had carious FPM, much higher than the caries prevalence of permanent teeth in children aged 12 in China nationwide (29%) (8). According to the report from Wang et al., (22) the caries prevalence of FPM among children aged 7, 8, and 9 in China nationwide were 12.18, 16.83, and 19.61%, respectively, much lower than that in this study. In addition, the prevalence of FPM in children aged 7–8 was higher than in children from Tehran (34.3%) (23). The complex etiology and various risk factors may contribute to the differences in prevalence between different regions or countries. Due to different positions and anatomical structures, the caries prevalence of mandibular FPM (45.1%) was significantly higher than that of the maxilla FPM (22.2%), which is consistent with other studies (21, 24).

In the previous study based on a nationwide sample from China, girls are supposed to have an earlier eruption of FPM than boys (22) and are likely to consume more sweets (25), leading to a higher caries prevalence. However, sex was not related to caries development in this study. Moreover, being the only child in the family did not increase caries risk, which was consistent with the findings of some researchers (23).

Compared with the high caries prevalence, the filling rate of FPM in this sample is also extremely low. It may be because parents have poor oral health awareness, mistakenly regard the FPM as the second primary molar, and think the FPM will be replaced with a new tooth. In this study, over 70% of parents did not know FPM. Additionally, many children aged 7–9 years may still have dental anxiety about cooperating with dentists, delaying the best time for the treatment (26). Therefore, improving parents' knowledge and intention to treat decayed teeth in time and preventing children's anxiety cannot be ignored besides caries prevention.

Dental sealant, being applied to a tooth surface to provide a physical barrier that prevents biofilm growth by blocking nutrition, is very effective for reducing the occurrence of pit and fissure caries (27). China started to apply the sealant to children aged 6–9 years for free in 2008, which has been popularized gradually in recent years. Xiangyun County, which eliminated poverty in September 2018 (28), started this program for second-grade schoolchildren in 2019. However, before the oral health professionals apply the sealant to the children, almost half of them have decayed FPM. It may imply that the program alone cannot reduce pre-existing high caries prevalence. The local government needs to improve the public awareness of protecting the six-molar when children are at an earlier age.

Besides individual risk factors, socioeconomic status can influence children's oral health. Parental education level may be associated with knowledge of beneficial oral health-related behaviors (29–31). Nevertheless, neither parental education nor family income affected the caries prevalence of FPM in this study. In addition to the main results, we found that children with a higher mother's education level or higher family income are more likely to receive fluoride varnish and brush their teeth twice a day. However, both the education level and family income were not associated with the consumption of desserts, which is a critical risk factor for caries. Furthermore, children's frequency of consuming candies increased with the family income and mothers' education level. Thus, family-related factors may have diverse effects on dental caries and associated factors, which reflect the complexity of caries etiology.

Brushing teeth twice daily with the appropriate method and fluoride toothpaste is the most acceptable and principal non-professional method for caries prevention and maintaining good oral hygiene (32). Moreover, a dose-response effect of fluoride toothpaste was demonstrated for children in a previous systematic review (33). In this study, over half of the children brushed their teeth twice daily but did not show lower DMFT, which was not consistent with the results reported by previous studies (34, 35). It may be because combined the brushing frequency, brushing duration, method, the use of fluoride toothpaste, the concentration of fluoride toothpaste and brushing effect, which were not investigated in this study, have a cumulative effect on caries prevention (36). Therefore, in future studies, we need to assess all the aspects of toothbrushing, especially the oral hygiene after toothbrushing, which the dental plaque indices can evaluate.

Fluoride prevents caries by reducing the solubility of enamel, promoting enamel remineralization, and affecting the metabolism of cariogenic bacteria (33). It is now recognized as the main factor responsible for the observed dramatic decline in caries prevalence worldwide (37). Fluoride varnish, one of the most common products for topical use, is recommended for children at high/moderate caries risk after the first primary tooth eruption (38). In this study, only 36.2% of the children had fluoride varnish experience and showed a significantly lower caries prevalence of FPM, indicating that popularizing fluoride varnish application is necessary for dental care promotion in local children. However, the frequency of fluoride varnish application was not recorded in this study, which will be included in our future research to assess fluoride varnish's effect on caries prevention.

Parental oral health knowledge was significantly associated with their children's dental caries (39). In general, most parents of this sample knew the importance of oral health, the role of sugar in caries development, and the necessity of treating caries in primary teeth. When parents did not know the necessity of treating decayed primary teeth, their children tended to have a much higher prevalence of FPM. Llena et al. (40) reported that caries in primary teeth was among the best predictors of caries in FPM. It is rational to deduce that children with primary teeth caries more or less have inappropriate oral health-related habits and behaviors, directly influencing the FPM health. Unfortunately, this study did not record the caries status of primary teeth. Thus, we could not verify the association between the caries status in primary teeth and FPM. We will include this point in future studies.

In 2019, China launched a “The Healthy China Initiative (2019–2030)” policy, in which one of the aims of caries prevalence programs in China is to reduce dental caries to <25% of 12-year-old children in 2030 (41). Based on the present study results, the local government of remote regions like Xiangyun needs to do much more to achieve this goal. In addition to applying a sealant to children aged 7–9, improving both parents' and children's oral-health awareness, establishing children's habit of periodic oral examinations, and promoting the treatment for decayed teeth in time in younger ages are also urgent for oral health promotion in this region.

## Conclusion

Caries prevalence of FPM among second-grade schoolchildren in Xiangyun was 47.6%, considerably higher than the average prevalence nationwide in China. Consuming desserts at least once a day, having no fluoride varnish application experience, worse parental perception of children's oral health status, and incorrect parental knowledge of whether decayed primary teeth need treatment are indicators of the high caries prevalence in this sample. The results of this study could provide a framework for ongoing and future public oral health programs and policy planning in Xiangyun, with particular attention to early preventive and therapeutic measures.

## Data availability statement

The raw data supporting the conclusions of this article will be made available by the authors, without undue reservation.

## Ethics statement

The studies involving human participants were reviewed and approved by the Ethics Committee of the People's Hospital of Xiangyun. Written informed consent to participate in this study was provided by the participants' legal guardian/next of kin.

## Author contributions

ML and XX analyzed the data and wrote the manuscript. QS, HZ, and FZ collected the data. GL conceived the idea, wrote the discussion section and revised the manuscript. All authors read and approved the final version of the manuscript prior to submission.

## Funding

This study received support from Research Program of People's Hospital of Xiangyun Affiliated to Dali University (DX2020SF02).

## Conflict of interest

The authors declare that the research was conducted in the absence of any commercial or financial relationships that could be construed as a potential conflict of interest.

## Publisher's note

All claims expressed in this article are solely those of the authors and do not necessarily represent those of their affiliated organizations, or those of the publisher, the editors and the reviewers. Any product that may be evaluated in this article, or claim that may be made by its manufacturer, is not guaranteed or endorsed by the publisher.
